# Evaluating the diagnostic accuracy of heat shock proteins and their combination with Alpha-Fetoprotein in the detection of hepatocellular carcinoma: a meta-analysis

**DOI:** 10.1186/s12876-024-03260-5

**Published:** 2024-05-21

**Authors:** Dan Xiang, Lifang Fu, Ying Yang, ChengJiang Liu, Yong He

**Affiliations:** 1 Department of Laboratory Medicine, Ya’an People’s Hospital, Yaan, 625000 China; 2 Department of Clinical Laboratory, the Second Affiliated Hospital, Army Medical University, Chongqing, 400037 China; 3 Department of General Medicine, Affiliated Anqing First People’s Hospital of Anhui Medical University, Anqing, 246000 China; 4 Department of Laboratory Medicine, West China Hospital, Sichuan University, Chengdu, 610041 China

**Keywords:** Hepatocellular carcinoma, Heat shock proteins, Alpha-fetoprotein, Meta-analysis

## Abstract

**Background:**

A growing body of research suggests that heat shock proteins (HSPs) may serve as diagnostic biomarkers for hepatocellular carcinoma (HCC), but their results are still controversial. This meta-analysis endeavors to evaluate the diagnostic accuracy of HSPs both independently and in conjunction with alpha-fetoprotein (AFP) as novel biomarkers for HCC detection.

**Methods:**

Pooled statistical indices, including sensitivity, specificity, diagnostic odds ratio (DOR), positive likelihood ratio (PLR), and negative likelihood ratio (NLR) with 95% confidence intervals (CI), were computed to assess the diagnostic accuracy of HSPs, AFP, and their combinations. Additionally, the area under the summary receiver operating characteristic (SROC) curve (AUC) was determined.

**Results:**

A total of 2013 HCC patients and 1031 control subjects from nine studies were included in this meta-analysis. The summary estimates for HSPs and AFP are as follows: sensitivity of 0.78 (95% CI: 0.69–0.85) compared to 0.73 (95% CI: 0.65–0.80); specificity of 0.89 (95% CI: 0.81–0.95) compared to 0.86 (95% CI: 0.77–0.91); PLR of 7.4 (95% CI: 3.7–14.9) compared to 5.1 (95% CI: 3.3–8.1); NLR of 0.24 (95% CI: 0.16–0.37) compared to 0.31 (95% CI: 0.24–0.41); DOR of 30.19 (95% CI: 10.68–85.37) compared to 16.34 (95% CI: 9.69–27.56); and AUC of 0.90 (95% CI: 0.87–0.92) compared to 0.85 (95% CI: 0.82–0.88). The pooled sensitivity, specificity, PLR, NLR, DOR and AUC were 0.90 (95% CI: 0.82–0.95), 0.94 (95% CI: 0.82–0.98), 14.5 (95% CI: 4.6–45.4), 0.11 (95% CI: 0.06–0.20), 133.34 (95% CI: 29.65–599.61), and 0.96 (95% CI: 0.94–0.98) for the combination of HSPs and AFP.

**Conclusion:**

Our analysis suggests that HSPs have potential as a biomarker for clinical use in the diagnosis of HCC, and the concurrent utilization of HSPs and AFP shows notable diagnostic effectiveness for HCC.

**Supplementary Information:**

The online version contains supplementary material available at 10.1186/s12876-024-03260-5.

## Background

Hepatocellular carcinoma (HCC) ranks second in cancer-related mortality rates globally, with approximately 90,000 new cases and over 800,000 cancer-related deaths reported worldwide in 2020, nearly half of which were in China [1–3]. Surgical resection and liver transplantation are the primary treatment options for early-stage HCC in current clinical practice [4, 5]. However, due to the absence of typical clinical symptoms and early warning signs, a significant proportion of patients are diagnosed at advanced stages, precluding potentially curative resection and resulting in a dismal 5-year survival rate [4–6]. Timely identification and effective intervention are essential factors in improving the prognosis of individuals diagnosed with HCC. Alpha-fetoprotein (AFP) serves as the primary screening and diagnostic biomarker for HCC but suffers from limited sensitivity, as approximately 40% of HCC patients have normal AFP levels, and only 20% of those with early-stage HCC exhibit elevated AFP levels [7, 8]. Hence, there is an urgent need for more accurate diagnostic biomarkers for early HCC detection.

Heat shock proteins (HSPs) are molecular chaperones that are ubiquitously present in archaea, fungi, and eukaryotes, exhibiting a high degree of conservation [9, 10]. These proteins are typically categorized into distinct groups based on their molecular weight, including HSP27, HSP40, HSP70, HSP90, HSP110, and chaperonins [11]. It has been observed that these proteins are integral in maintaining protein homeostasis by facilitating the proper folding and unfolding of proteins, and they also play crucial roles in the regulation of apoptosis [12–15]. Initially identified as intracellular chaperones, HSPs have also been detected in the extracellular environment. In extracellular spaces, HSPs have been linked to tumor invasiveness, tumor immunity, resistance to anti-tumor treatments, and unfavorable clinical outcomes, thereby playing a significant role in tumor progression and development [16–18]. Elevated levels of HSP expression have been observed in various human malignancies, including HCC, colorectal cancer, cervical cancer, breast cancer, prostate cancer, and lung cancer [19]. Several studies have demonstrated that HSPs are potential biomarkers for cancer diagnosis and prognosis [19–21]. Recently, a few studies estimated the diagnostic value of HSPs for detecting HCC, but the diagnostic accuracies are inconsistent and even conflicting [22–30]. Thus, we conducted this systematic review and meta-analysis to evaluate the diagnostic efficacy of HSPs for detecting HCC. Additionally, we also compared the diagnostic value of HSPs, AFP, and the combination of both based on the pooled statistical indicators.

## Methods

This meta-analysis was performed based on the Preferred Reporting Items for Systematic Reviews and Meta-analysis (PRISMA) statement [31], and the PRISMA checklist is shown in the Supplementary Table 1. The study protocol was registered on PROSPERO with registration number CRD42023442862.

### Literature search

Unrestricted by language, year of publication, or publication status, a comprehensive search was conducted for relevant studies on the diagnostic utility of HSPs in detecting HCC up to January 1, 2024. This search encompassed multiple databases including PubMed, Cochrane Library, Web of Science, Embase, EBSCO, Scopus, Chinese National Knowledge Infrastructure (CNKI), Wan Fang, and VIP. Two researchers independently performed the search using specified search terms outlined in Supplementary Table 2, which included keywords such as "heat shock proteins" and "hepatocellular carcinoma." Additional eligible articles were identified by manually searching the references of included studies.

### Inclusion and exclusion criteria

Two authors conducted a screening of relevant articles by reviewing titles and abstracts, followed by a thorough examination of the full-text based on predetermined inclusion and exclusion criteria. Any discrepancies were resolved through discussion with a third author to achieve a final consensus. The inclusion criteria for the present meta-analysis were as follows: 1) articles that evaluated the diagnostic value of serum or plasma HSPs in detection of HCC; 2) the HSPs were tested by enzyme-linked immunosorbent assay (ELISA); 3) the diagnosis of HCC was made on the basis of histopathology; 4) the sample size of patients and controls, true positive (TP), false positive (FP), true negative (TN) and false negative (FN) were reported or could be calculated; 5) study was published in English or Chinese with full-text available. In addition, the exclusion criteria were applied: Letters, reviews, conference abstracts, animal experiments, fundamental research, case reports and duplicated reports; sample size of case and control patients was less than 20.

### Data extraction and quality assessment

Two authors independently extracted the following data: first author, publication year, country, sample size of patients and controls, control populations, cut-off values, assay method of the biomarkers, TP, FP, TN and FN.

The quality of included studies is assessed using Quality Assessment of Diagnostic Accuracy Studies 2 (QUADAS-2) tool, which is reliable for quality assessment of diagnostic accuracy tests [32]. The QUADAS-2 tool comprises four domains: patient selection, index test, reference standard, and flow and trimming. Each domain is evaluated for risk of bias, with the first three domains also assessed for applicability. Key areas crucial for quality assessment include participant selection, blinding, and missing data. Risk of bias and applicability concerns are rated as "high," "unclear," or "low" by the QUADAS-2. Disagreements were resolved through discussion with a third investigator to achieve a final consensus in data extraction and quality assessment.

### Statistical analysis

The meta-analysis was performed using Stata 16.0 and Review Manager 5.3 software. Threshold effect was assessed by Spearman correlation coefficient and *P*- value. *P* < 0.05 indicated the existence of a threshold effect. Heterogeneity induced by non-threshold effect was estimated using the* I*^2^ value and Cochran’s Q test, with* I*^2^ > 50% and *P* < 0.05 suggesting significant heterogeneity. When *P* > 0.05 and *I*^2^ < 50%, a fixed-effect model was used for meta-analysis, while a random-effect model was used. Pooled sensitivity, specificity, diagnostic odds ratio (DOR), positive likelihood ratio (PLR), and negative likelihood ratio (NLR) with 95% confidence interval (CI) were calculated and presented in the form of forest plots. Summary receiver operator characteristic (SROC) curves were plotted, and the area under curve (AUC) was calculated. The AUC was used for grading the overall diagnostic accuracy of HSPs and AFP in HCC. A diagnostic tool is described as perfect if AUC is 1.00, excellent if the AUC is greater than 0.90, good if it is greater than 0.80, moderate if it is less than 0.80 [33]. Subgroup analysis was conducted to further explore the source of heterogeneity. Furthermore, we planned to use funnel plots to assess the publication bias if there were greater than or equal to 10 included studies [34].

## Results

### Characteristics and quality evaluation of included studies

**Fig. 1:**
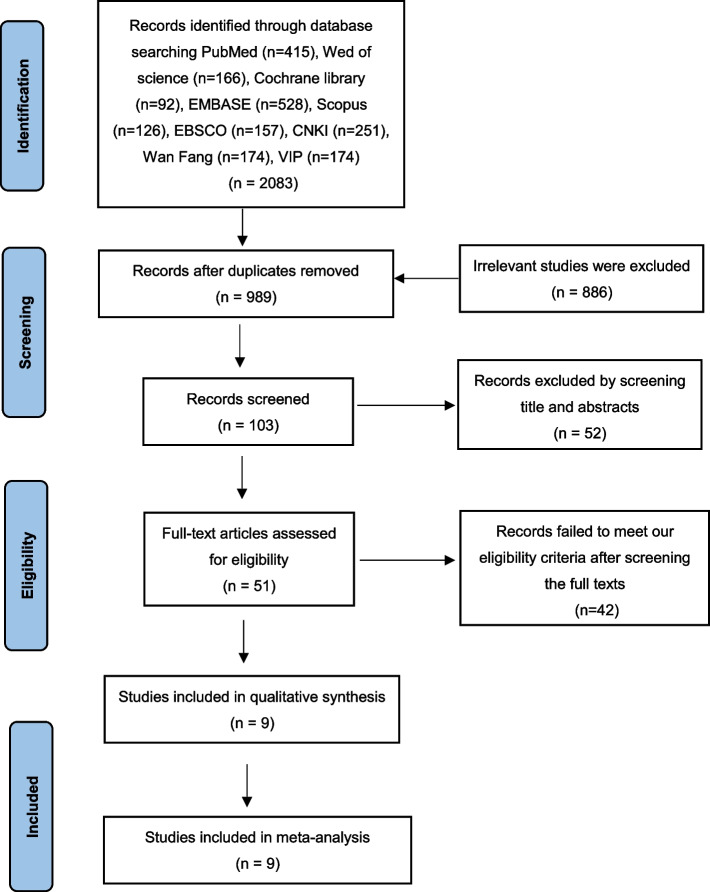
The flow chart of study selection

As shown in Fig. 1, a total of 2083 relevant articles were retrieved based on our search strategy. Nine articles were enrolled in our meta-analysis, including 2013 patients and 1031 controls. All the studies were published between 2011 and 2021, with eight studies from China and one study from Italy [22–30]. The basic characteristic of included studies is shown in Table 1.

**Table 1:** Main characteristics and diagnostic performance of individual studies

Study	Year	Country	Tumor maker	Test method	Cut-off value	TP	FP	FN	TN
Zhang et al. [22]	2019	China	HSP 90α	ELISA	271.595 ng/ml	86	2	4	88
Chen et al. [23]	2018	China	HSP 90α	ELISA	86.94 ng/ml	79	49	23	101
Tang et al. [24]	2020	China	HSP 90α	ELISA	81.65 ng/ml	297	12	112	167
			AFP	ECLIA	8.78 ng/ml	259	10	150	169
Gabriella et al. [25]	2013	Italy	HSP 27	ELISA	456.5 pg/ml	50	22	21	58
			AFP	CLIA	10.1 ng/ml	50	26	21	54
Li et al. [26]	2016	China	HSP 27	ELISA	66.5 pg/ml	37	0	13	30
			AFP	CLIA	20.4 ng/ml	35	1	15	29
Wei et al. [27]	2020	China	HSP 90α	ELISA	69.1 ng/mL	442	22	217	208
			AFP	ECLIA	5.38 ng/ml	535	14	124	216
			HSP 90α + AFP	/	/	566	4	93	226
Han et al. [28]	2021	China	HSP 90α	ELISA	76.46 ng/ml	37	3	10	37
			AFP	CLIA	/	45	10	2	30
			HSP 90α + AFP	/	/	45	1	2	39
Fu et al. [29]	2017	China	HSP 90α	ELISA	73.23 ng/mL	453	16	56	149
			AFP	ELISA	6.171 ng/ml	391	27	118	129
			HSP 90α + AFP	/	/	479	17	30	139
Wang et al. [30]	2011	China	HSP 27	ELISA	81.63 ng/ml	45	18	31	49
			AFP	CLIA	25ug/L	46	12	30	55
			HSP 27 + AFP	/	/	60	6	16	19

*HSP* heat shock protein, *95%CI* 95% confidence interval, *AUC* area under curve, *TP* true positive, *FP* false positive, *FN* false negative, *TN* true negative, *CLIA* chemiluminescence immunoassay, *ECLIA* electrochemiluminescence immunoassay, *ELISA *enzyme-linked immunosorbent assay

**Fig. 2:**
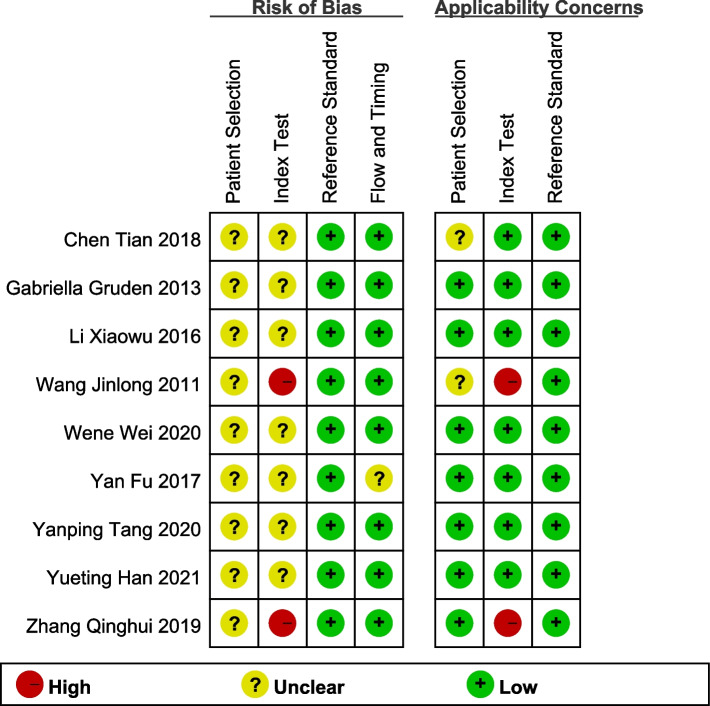
Risk of bias assessment for each included study

The assessment of study quality was carried out using the QUADAS-2 tool, with the results summarized in Fig. 2. Regarding the patient selection domain, all studies were deemed to have an unclear risk of bias due to their case–control design [22–30]. Within the index test domain, two studies showed a high risk of bias [22, 30], with the remaining studies rated as unclear risk of bias [23–29]. None of the studies were found to have a high risk of bias in the reference standard or flow and timing domains. Further details of the assessment can be found in Supplementary Table 3.

### Meta-analysis of diagnostic efficacy

**Fig. 3:**
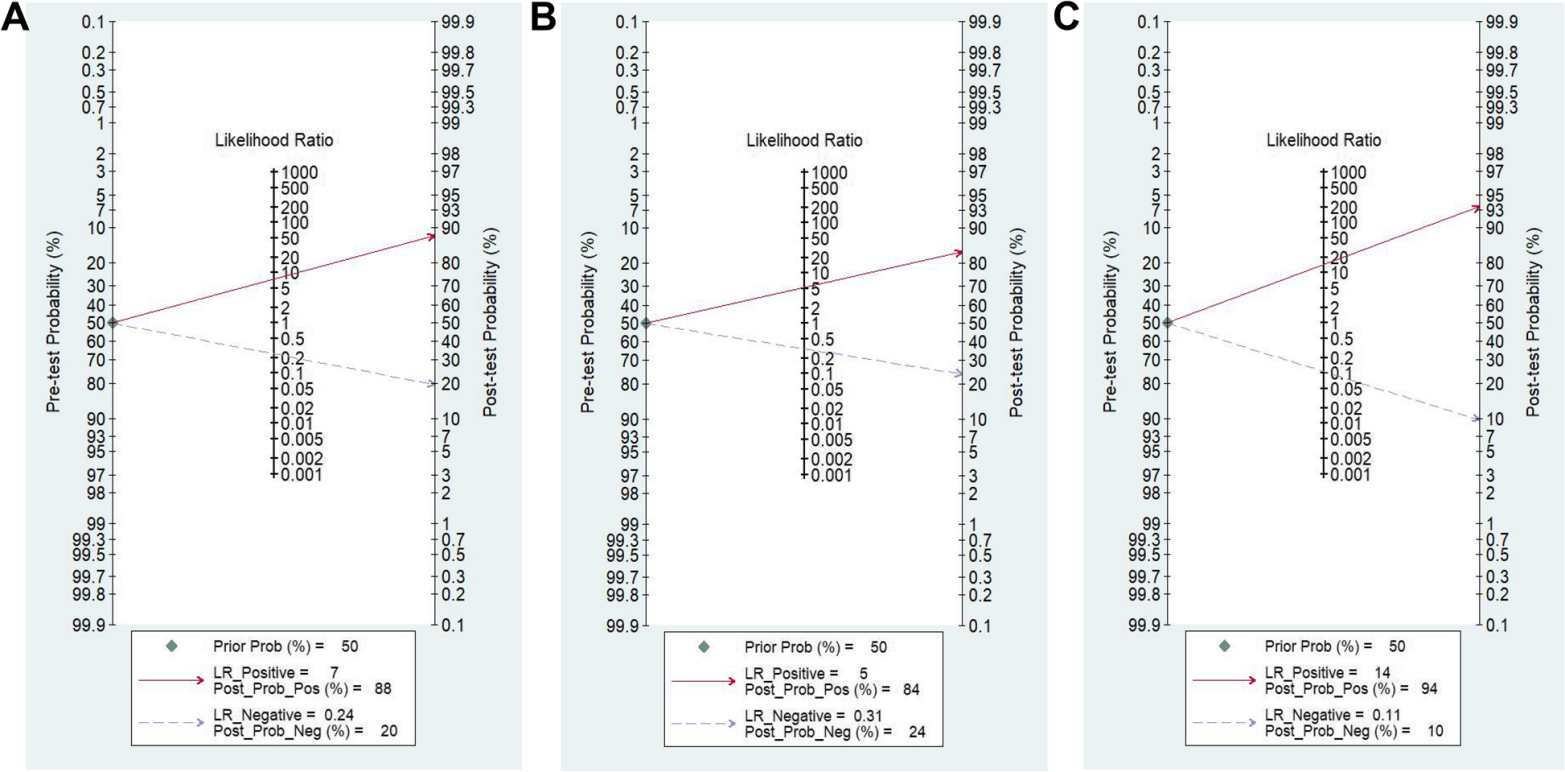
Fagan diagram assessing the overall diagnostic value of heat shock proteins (**A**), alpha-fetoprotein (**B**) and heat shock proteins combination with alpha-fetoprotein (**C**) for hepatocellular carcinoma

Nine studies were conducted to evaluate the diagnostic value of HSPs in HCC. Given the significant heterogeneity (sensitivity,* I*^2^ = 93.03% and specificity, *I*^2^ = 93.08%) among these studies, the random-effects model was utilized to synthesize the data, revealing no threshold effect (Spearman correlation coefficient: 0.59, *P *= 0.35). As depicted in Table 2, the pooled sensitivity and specificity were 0.78 (95% CI: 0.69- 0.85, *I*^2^ = 93.03%) and 0.89 (95% CI: 0.81- 0.95, *I*^2^ = 93.08%), respectively. The PLR and NLR were 7.4 (95% CI: 3.7- 14.9, *I*^2^ = 90.58%) and 0.24 (95% CI: 0.16- 0.37, *I*^2^ = 92.76%), respectively. The DOR of pooled studies was 30.19 (95% CI: 10.68- 85.37, *I*^2^ = 100%), and the AUC for SROC was 0.90 (95% CI: 0.87- 0.92), indicating good overall accuracy of HSPs for HCC. A Fagan nomogram was constructed to visually represent the diagnostic accuracy, demonstrating an increase in probability to 88% in patients with HSPs and a decrease to 20% in those without HSPs (Fig. 3A).

**Table 2:** Summary of the pooled diagnostic indices of heat shock proteins, alpha-fetoprotein and combination of both for hepatocellular carcinoma

Summary		HSPs	AFP	Combination
Sensitivity	95%CI	0.78 (0.69- 0.85)	0.73 (0.65- 0.80)	0.90 (0.82- 0.95)
	*I*^2^	93.03%	84.36%	90.04%
Specificity	95%CI	0.89 (0.81- 0.95)	0.86 (0.77- 0.91)	0.94 (0.82- 0.98)
	*I*^2^	93.08%	86.18%	92.53%
DOR	95%CI	30.19 (10.68–85.37)	16.34 (9.69- 27.56)	133.34 (29.65- 599.61)
	*I*^2^	100%	99.92%	100%
PLR	95%CI	7.4 (3.7- 14.9)	5.1 (3.3- 8.1)	14.5 (4.6- 45.4)
	*I*^2^	90.58%	71.66%	87.73%
NLR	95%CI	0.24 (0.16- 0.37)	0.31 (0.24- 0.41)	0.11 (0.06- 0.20)
	*I*^2^	92.76%	74.05%	89.95%
AUC	95%CI	0.90 (0.87- 0.92)	0.85 (0.82- 0.88)	0.96 (0.94- 0.98)

*HSPs* Heat shock proteins, *AFP* alpha-fetoprotein, *HCC* hepatocellular carcinoma, *CI* confidence interval, *AUC* area under the SROC curve, *DOR* diagnostic odds ratio, *PLR* positive likelihood ratio, *NLR* negative likelihood ratio

In all of these studies, seven specifically investigated the diagnostic accuracy of AFP for HCC. The analysis revealed substantial heterogeneity (sensitivity,* I*^2^ = 84.36% and specificity, *I*^2^ = 86.18%) among the included studies, with no evidence of a threshold effect (Spearman correlation coefficient = -0.58, *P* = 0.33). The pooled sensitivity was 0.73 (95% CI: 0.65- 0.80, *I*^*2*^ = 84.36%), specificity was 0.86 (95% CI: 0.77- 0.91, *I*^2^ = 86.18%), PLR was 5.1 (95% CI: 3.3- 8.1, *I*^2^ = 71.66%), NLR was 0.31 (95% CI: 0.24- 0.41, *I*^2^ = 74.05%), and DOR was 16.34 (95% CI: 9.69- 27.56, *I*^2^ = 99.92%), respectively. The AUC for SROC was 0.85 (95% CI: 0.82- 0.88) (Table 2). Utilizing the Fagan plot, the likelihood of HCC diagnosis increased to 84% in patients with elevated AFP levels, while decreasing to 24% in those without elevated AFP levels, based on 50% of patients being diagnosed with HCC (Fig. 3B).

Furthermore, four studies assessed the diagnostic accuracy of the combination of HSPs and AFP, the pooled sensitivity was 0.90 (95%CI: 0.82- 0.95, *I*^2^ = 90.04%), specificity was 0.94 (95%CI: 0.82- 0.98, *I*^2^ = 92.53%), PLR was 14.5 (95%CI: 4.6- 45.4, *I*^2^ = 87.73%), NLR was 0.11 (95%CI: 0.06- 0.20, *I*^2^ = 89.85%), DOR was 133.34 (95%CI: 29.65- 599.61, *I*^2^ = 100%), and the corresponding AUC was 0.96 (95%CI: 0.94- 0.98) (Table 2). Additionally, the Fagan plot demonstrated that the combination of HSPs and AFP could increase the post-test probability to 94% in patients and decrease the post-test probability to 10% in patients with a pre-test probability of 50% (Fig. 3C), indicating a high overall accuracy of the combination of HSPs and AFP for detecting HCC.

### Subgroup analysis

To investigate the heterogeneity resulting from the non-threshold effect, subgroup analysis was conducted based on various factors including control population, case sample size (≥ 100 or < 100), HSPs type, and specimen type. The findings of the subgroup analysis are presented in Table 3. None of the covariates mentioned above were found to contribute to heterogeneity in the HSPs group. However, in the AFP group, low heterogeneity was observed in the healthy control population group and the case sample size ≥ 100 group, with *I*^2^ values of 20.8% and 13.2%, respectively. This suggests that differences in control population and case sample size may be the underlying source of heterogeneity.

**Table 3:** Subgroup analysis of the included studies

Subgroup	No	DOR (95%CI)	Effect model	Heterogeneity	
				*I*^*2*^	*P*
HSPs					
Control population					
HCC vs Healthy	4	19.64(7.77, 49.65)	random	76.70%	0.005
HCC vs non-HCC	5	31.96(7.74, 131.99)	random	94.50%	0.000
Case sample size					
≥ 100	4	24.70(9.56, 63.83)	random	91.30%	0.000
< 100	5	35.54(6.54, 192.96)	random	90.80%	0.000
HSPs Type					
HSP90α	6	41.58(16.25, 106.40)	random	90.50%	0.000
HSP27	3	7.37(2.53, 21.41)	random	69.10%	0.039
Specimen type					
Serum	3	7.37(2.53, 21.41)	random	69.10%	0.039
Plasma	6	41.58(16.25, 106.40)	random	90.50%	0.000
AFP					
Control population					
HCC vs Healthy	3	30.93(14.59, 65.59)	random	20.80%	0.283
HCC vs NHCC	4	11.49(5.48, 24.08)	random	81.50%	0.001
Case sample size					
≥ 100	3	20.28(14.38, 28.59)	random	13.20%	0.316
< 100	4	14.73(4.68, 46.34)	random	75.50%	0.000

*NO*. number of included studies, *HSPs* heat shock proteins, *AFP* alpha-fetoprotein, *HCC* hepatocellular carcinoma, *non-HCC* including health checkups and patients with benign liver diseases, *DOR* diagnostic odds ratio, *CI* confidence interval

### Publication bias

Considering the small sample size (n < 10) in our meta-analysis, funnel plot analysis was not applicable for the determination of publication bias.

## Discussion

HCC poses a significant challenge to public health due to its high incidence and mortality rates, with a 5-year overall survival rate of less than 10% [1, 2, 5]. Prompt diagnosis plays a pivotal role for improving outcomes for individuals with HCC. AFP stands as the most extensively studied diagnostic biomarker for HCC, but its effectiveness is limited, with sensitivities ranging from 0.39 to 0.65 and specificities ranging from 0.76 to 0.97 [35, 36]. This hinders the utility of AFP in the diagnosis of HCC. In recent studies, alternative biomarkers such as des-γ-carboxy prothrombin (DCP), Glypican-3 (GPC-3), and Golgi protein 73 (GP73) have been utilized for the detection of HCC [37]. Zhao et al. conducted a meta-analysis to evaluate the diagnostic efficacy of GPC-3, resulting in a combined sensitivity of 0.59 and specificity of 0.93 in serum GPC-3 for HCC detection [38]. Another recent meta-analysis examined the diagnostic utility of GP73, revealing combined sensitivity, specificity, and AUC values of 0.79, 0.85, and 0.88, respectively [39]. Moreover, previous studies have reported that DCP exhibits sensitivities and specificities within the ranges of 0.61 to 0.77 and 0.70 to 0.82 [40, 41]. Despite advancements in diagnostic techniques over recent years, the timely detection of HCC continues to present challenges [42, 43]. So, there is a need to identify supplementary biomarkers that are closely associated with the progression of HCC in order to enhance the accuracy of diagnosis and treatment.

HSPs are ubiquitously present in biological cells [9]. Oncoproteins often rely on elevated levels of HSPs to sustain their functionality, with tumor cells exhibiting notably higher levels of HSPs compared to their normal counterparts in a range of cancers such as lung, colorectal, prostate cancers, and HCC [44–48]. Extensive research has been conducted in recent decades to elucidate the relationship between HSPs and tumor occurrence and progression, mainly focusing on HSP27, HSP70, and HSP90 in HCC [49–55]. HSP27, a member of the small HSP family, plays a critical role in the invasion and metastasis of HCC by binding to the N-terminus of AKT and connecting MAPK activated protein kinase 2 (MK2) to AKT, thereby regulating the synthesis of integrins α- Expression of 7 (ITGA7) and matrix metalloproteinase 2 (MMP2) [49, 50]. Zhang et al. demonstrated that elevated levels of HSP27 are associated with increased metastasis of HCC and established HSP27 as a valuable prognostic indicator for HCC outcomes [50]. Additionally, in the hypoxic and stressed tumor microenvironment of early-stage HCC, HSP70 is notably upregulated and may serve as a sensitive marker for precancerous lesions. Furthermore, in advanced stages of HCC, HSP70 expression is positively correlated with tumor size, portal and microvascular invasion, and inversely correlated with disease-free survival [51–54]. HSP90, a pivotal molecular chaperone, plays a crucial role in binding to the kinase SRPK2 and controlling the selective splicing of Numb PRR isoforms, ultimately facilitating HCC proliferation, invasion, and metastasis [55]. Overall, HSPs have a notable influence on the development of HCC, and may serve as a potential diagnosis biomarker for HCC.

In this meta-analysis, we systematically evaluated the diagnostic accuracy of HSPs, AFP, and the combination of HSPs with AFP in distinguishing HCC patients from non-HCC controls. To the best of our knowledge, this is the first systematic review and meta-analysis to estimate the diagnostic accuracy of HSPs and the combination of HSPs with AFP for HCC. We included nine studies with a total of 2013 patients in our analysis. The results of our study indicate that AFP exhibited a sensitivity of 0.73 and specificity of 0.86, with an AUC of 0.85. In comparison, HSPs demonstrated higher sensitivity (0.78), specificity (0.89), and AUC (0.90), suggesting that HSPs possess favorable diagnostic capabilities for distinguishing HCC patients from non-HCC controls. Our study supports the use of HSPs as an alternative to AFP for assessing HCC.

Due to the limitations of single biomarkers in accurately determining both sensitivity and specificity, the combination of multiple biomarkers holds significant potential for improving the diagnosis of HCC. A recent meta-analysis revealed that the combination of AFP and DCP can enhance diagnostic accuracy, with pooled sensitivity and specificity rates of 0.82 and 0.85, respectively, and AUC of 0.90 [56]. Additionally, Zhao et al. demonstrated that combining GPC-3 and AFP resulted in a pooled sensitivity of 0.71 and specificity of 0.91, with an AUC of 0.85 [38]. In a separate meta-analysis conducted in 2020, the combined use of AFP, AFP-L3, and DCP demonstrated a high diagnostic efficacy in discriminating HCC, with a pooled sensitivity of 0.88, specificity of 0.79, and an AUC of 0.91 [57]. In the current investigation, we evaluated the diagnostic value of combining HSPs with AFP, revealing that this combined approach significantly improved diagnostic accuracy, resulting in a sensitivity of 0.90, specificity of 0.94, and an AUC of 0.96. Our findings confirmed that the combination of HSPs and AFP has better diagnostic performance than other biomarkers alone or combination, and may also further provide a new insight into the diagnosis of HCC patients. Further investigation through clinical trials is necessary to validate the potential utility of HSPs, either in combination or alone, as a biomarker for diagnosing HCC.

There was considerable heterogeneity between the included studies in our meta-analysis. Initially, we identified the threshold effect through the Spearman correlation analysis, and none of the results exhibited the threshold effect*.* Subsequently, a subgroup analysis was conducted to explore the potential sources of heterogeneity. As shown in Table 3, the subgroup results of HSPs suggested that the *I*^2^ of most subgroups was still more than 50%, indicating that these factors were not the source of heterogeneity. For AFP, the subgroup results of AFP suggested that the *I*^2^ of healthy control population and case sample size ≥ 100 group was 20.8% and 13.2%, respectively, indicating the different control population and case sample size may be the source of heterogeneity.

Several limitations need to be acknowledged in this study. Firstly, a significant proportion (88.89%) of the studies included in the analysis originated from China, potentially restricting the generalizability of our findings. This skewed representation may be attributed to the high incidence of new cancer cases and related deaths in China [1, 2]. Secondly, the level of evidence was low, as all the included studies were case–control, which may introduce the potential for bias. Thirdly, inconsistencies in the cut-off values used across the included studies could introduce variability in the results. Therefore, as a biomarker, HSPs still need to be tested for detecting HCC in future studies to analyze the suitable cut-off value. Additionally, the diagnostic value of HSPs for HCC patients at varying pathological stages was not assessed in this study due to the absence of original research data, highlighting the need for further investigation on this matter. Furthermore, significant heterogeneity persisted in certain subgroups, emphasizing the necessity for additional research in this area.

## Conclusions

In summary, our meta-analysis indicates that HSPs serve as accurate biomarkers suitable for clinical use in the diagnosis of HCC, and the combination of HSPs and AFP significantly enhances diagnostic value compared to HSPs or AFP alone. However, further research studies characterized by rigorous methodology, substantial sample sizes, and collaboration across multiple centers are imperative to gather more conclusive evidence regarding the diagnostic utility of HSPs and the combined use of HSPs and AFP in the early detection of HCC.

### Supplementary Information

Supplementary Material 1.

Supplementary Material 2.

Supplementary Material 3.

## Data Availability

All data and materials during this study are presented within the manuscript.

## Abbreviations

HSPs: Heat shock proteins
HCC: Hepatocellular carcinoma
AFP: Alpha-fetoprotein
DOR: Diagnostic odds ratio
PLR: Positive likelihood ratio
NLR: Negative likelihood ratio
95%CI: 95% Confidence interval
SROC: Summary receiver operator characteristic
AUC: Area under curve
