# Supplementary material for: Evaluating the diagnostic accuracy of heat shock proteins and their combination with Alpha-Fetoprotein in the detection of hepatocellular carcinoma: a meta-analysis
Source: BMC Gastroenterol. 2024 May 21;24:178. doi: 10.1186/s12876-024-03260-5 (PMC11110180; doi:10.1186/s12876-024-03260-5)
Supplement: Supplementary file 2 — Supplementary Material 2. [file 12876_2024_3260_MOESM2_ESM.docx]

**Supplementary Table 2** Search strategy

| **PubMed** | |
| --- | --- |
| **No.** | **Search items** |
| #1 | ((((heat shock proteins [Title/Abstract]) OR (stress proteins [Title/Abstract])) OR (heat shock protein [Title/Abstract])) OR (stress protein [Title/Abstract])) OR (HSPs [Title/Abstract]) |
|  |  |
| #2 | (((((((((carcinomas, hepatocellular[Title/Abstract]) OR (hepatocellular carcinoma[Title/Abstract])) OR (hepatocellular cancer[Title/Abstract])) OR (hepatocellular tumor[Title/Abstract])) OR (hepatocellular neoplasm[Title/Abstract])) OR (liver cell carcinoma[Title/Abstract])) OR (liver cell cancer[Title/Abstract])) OR (liver cell neoplasm[Title/Abstract])) OR (liver cell tumor[Title/Abstract])) OR (HCC[Title/Abstract]) |
|  |  |
|  |  |
|  |  |
|  |  |
| #3 | #1 AND #2 |
