# Supplementary material for: Evaluating the diagnostic accuracy of heat shock proteins and their combination with Alpha-Fetoprotein in the detection of hepatocellular carcinoma: a meta-analysis
Source: BMC Gastroenterol. 2024 May 21;24:178. doi: 10.1186/s12876-024-03260-5 (PMC11110180; doi:10.1186/s12876-024-03260-5)
Supplement: Supplementary file 3 — Supplementary Material 3. [file 12876_2024_3260_MOESM3_ESM.docx]

**Supplementary Table 3** Detailed details for the assessment of bias risk and applicability risk in included studies using QUADAS-2.

| Included studies | | Li et al ^[26]^ | Gabriella et al ^[25]^ | Wang et al ^[30]^ | Wei et al ^[27]^ | Fu et al ^[29]^ | Tang et al ^[24]^ | Han et al ^[28]^ | Zhang et al ^[22]^ | Chen et al ^[23]^ |
| --- | --- | --- | --- | --- | --- | --- | --- | --- | --- | --- |
|  |  |  |  |  |  |  |  |  |  |  |
| Patient selection | Iconic question 1 | Yes | Yes | Unclear | Yes | Yes | Yes | Yes | Yes | Yes |
|  | Iconic question 2 | No | No | No | No | No | No | No | No | No |
|  | Iconic question 3 | Yes | Yes | Yes | Yes | Yes | Yes | Yes | Yes | Unclear |
|  | Risk level | UR | UR | UR | UR | UR | UR | UR | UR | UR |
|  | Clincal applicability | LC | LC | UC | LC | LC | LC | LC | LC | UC |
| Index Text | Iconic question 1 | Unclear | Yes | Unclear | Yes | Yes | Yes | Yes | Unclear | Yes |
|  | Iconic question 2 | Yes | No | No | No | No | No | No | No | No |
|  | Risk level | UR | UR | HR | UR | UR | UR | UR | HR | UR |
|  | Clincal applicability | LC | UC | HC | UC | UC | UC | UC | HC | UC |
| Reference standard | Iconic question 1 | Yes | Yes | Yes | Yes | Yes | Yes | Yes | Yes | Yes |
|  | Iconic question 2 | Yes | Yes | Yes | Yes | Yes | Yes | Yes | Yes | Yes |
|  | Risk level | LR | LR | LR | LR | LR | LR | LR | LR | LR |
|  | Clincal applicability | LC | LC | LC | LC | LC | LC | LC | LC | LC |
| Flow and triming | Iconic question 1 | Yes | Yes | Yes | Yes | Yes | Yes | Yes | Yes | Yes |
|  | Iconic question 2~3 | Yes | Yes | Yes | Yes | Yes | Yes | Yes | Yes | Yes |
|  | Iconic question 4 | Yes | Yes | Yes | Yes | No | Yes | Yes | Yes | Yes |
|  | Risk level | LR | LR | LR | LR | UR | LR | LR | LR | LR |

LR: low risk; HR: high risk; UR: unclear risk; LC: low concern; HC: high concern; UC:unclear concern.
